# Supplementary material for: Gene count normalization in single-cell imaging-based spatially resolved transcriptomics
Source: Genome Biol. 2024 Jun 12;25:153. doi: 10.1186/s13059-024-03303-w (PMC11167774; doi:10.1186/s13059-024-03303-w)
Supplement: Supplementary file 1 — Additional file 1: Figures S1-14 [file 13059_2024_3303_MOESM1_ESM.docx]

**Supplementary Figures**

**Figure S1. Gene expression of brain anatomical regions in full and simulated MERFISH gene panel**

**A.** Heatmap showing scaled mean gene expression of 481 genes in full MERFISH panel for the four analyzed brain regions.

**B.** Heatmap showing scaled mean gene expression of the 100 genes in 100-gene gene panel skewed towards ventricle brain region for the four analyzed brain regions.

**C.** Heatmap showing scaled mean gene expression of the 100 genes in 100-gene gene panel skewed towards habenula brain region for the four analyzed brain regions.

**D.** Heatmap showing scaled mean gene expression of the 100 genes in 100-gene gene panel skewed towards fiber tract brain regions for the four analyzed brain regions.

**E.** Heatmap showing scaled mean gene expression of the 100 genes in 100-gene gene panel skewed towards dentate gyrus brain regions for the four analyzed brain regions.

**F.** Heatmap showing scaled mean gene expression of the 100 genes in 1100-gene non-skewed gene panel for the four analyzed brain regions.

**G.** Gene panel skew of all simulated and original full MERFISH gene panels.

In all panels, purple, green, blue, and red indicate the ventricles, habenula, fiber tracts, and dentate gyrus regions, respectively.

**Figure S2: Scaling factors with MERFISH gene panels skewed towards the habenula, fiber tract, and dentate gyrus regions**

**A.** For a coronal section of the mouse brain assayed by MERFISH, scatterplots of log_10_(scaling factors) for each cell based on different normalization methods (library size normalization, DESeq2 normalization, TMM normalization, and cell volume normalization) for the full gene panel versus the habenula region-skewed gene panel. Cells within the habenula brain region are shown in green. Cells in the fiber tract, ventricle, and dentate gyrus brain regions are shown in grey. Red line indicates x=y.

**B.** Scatterplots of log_10_(scaling factors) for each cell based on different normalization methods (library size normalization, DESeq2 normalization, TMM normalization, and cell volume normalization) for the full gene panel versus the fiber tract region-skewed gene panel. Cells within the fiber tract brain regions are shown in blue. Cells in the habenula, ventricle, and dentate gyrus brain regions are shown in grey. Red line indicates x=y.

**C.** Scatterplots of log_10_(scaling factors) for each cell based on different normalization methods (library size normalization, DESeq2 normalization, TMM normalization, and cell volume normalization) for the full gene panel versus the dentate gyrus region-skewed gene panel. Cells within the dentate gyrus brain region are shown in red. Cells in the habenula, ventricle, and fiber tract brain regions are shown in grey. Red line indicates x=y.

**D.** Root mean squared error in scaling factors after each normalization method between the different region-skewed gene panels and the full gene panel.

**E.** Boxplot of Pearson correlation coefficients (*r*) across genes for normalized gene expression magnitudes with the full gene panel versus the habenula (left), fiber tract (middle), and dentate gyrus (right) region-skewed gene panels across different normalization methods evaluated.

**Figure S3. Differential gene expression p-values with full and region-skewed MERFISH gene panels**

**A.** Wilcoxon rank sum test -log_10_(p-values) adjusted for multiple hypothesis testing for one region-vs-all differential gene expression test with normalized gene expression from the full gene panel versus the habenula region-skewed gene panel. Green indicates habenula-vs-all tests, grey indicates other region-vs-all tests. Red line indicates x=y.

**B.** Wilcoxon rank sum test -log_10_(p-values) adjusted for multiple hypothesis testing for one region-vs-all differential gene expression test with normalized gene expression from the full gene panel versus the fiber tract region-skewed gene panel. Blue indicates fiber tract-vs-all tests, grey indicates other region-vs-all tests. Red line indicates x=y.

**C.** Wilcoxon rank sum test -log_10_(p-values) adjusted for multiple hypothesis testing for one region-vs-all differential gene expression test with normalized gene expression from the full gene panel versus the dentate gyrus region-skewed gene panel. Red indicates dentate gyrus-vs-all tests, grey indicates other region-vs-all tests. Red line indicates x=y.

In all panels, green, blue, and red indicate habenula, fiber tract, and dentate gyrus-specific genes, respectively.

**D.** Differential gene expression false positive rates after each normalization method, taking differential gene expression results with the full gene panel to be ground truth, for each region-skewed gene panel.

**E.** Differential gene expression false negative rates after each normalization method, taking differential gene expression results with the full gene panel to be ground truth, for each region-skewed gene panel.

**Figure S4. Gene log_2_ fold change with full and region-skewed MERFISH gene panels**

**A.** One region-vs-all gene fold change with normalized gene expression from the full gene panel versus the habenula region-skewed gene panel. Green indicates habenula-vs-all comparisons, grey indicates other region-vs-all comparisons.

**B.** One region-vs-all gene fold change with normalized gene expression from the full gene panel versus the fiber tract region-skewed gene panel. Blue indicates habenula-vs-all comparisons, grey indicates other region-vs-all comparisons.

**C.** One region-vs-all gene fold change with normalized gene expression from the full gene panel versus the dentate gyrus region-skewed gene panel. Red indicates habenula-vs-all comparisons, grey indicates other region-vs-all comparisons.

In all panels, black lines indicate 0 log_2_ fold change, grey lines indicate 0.5 log_2_ fold change, red line indicates x=y.

**D.** Gene expression fold change switched positive after each normalization method, taking differential gene expression results with the full gene panel to be ground truth, for each region-skewed gene panel.

**E.** Gene expression fold change switched negative rates after each normalization method, taking differential gene expression results with the full gene panel to be ground truth, for each region-skewed gene panel.

**Figure S5. Impact of gene count normalization methods with region-skewed gene panels in STARmap PLUS mouse brain im-SRT data**

**A.** For a coronal section of the mouse brain assayed by STARmap, heatmaps showing scaled mean gene expression of the 100 genes in 100-gene gene panel skewed towards ventricle (left), fiber tract (middle), and dentate gyrus (right) brain regions for the three analyzed brain regions.

**B.** Gene panel skew of all simulated and original full STARmap PLUS gene panels.

**C.** Scatterplots of log_10_(scaling factors) with library size normalization for each cell for ventricle (left), fiber tract (middle), and dentate gyrus (right) region-skewed gene panels versus full gene panel. Cells in the ventricle, fiber tract, and dentate gyrus regions are shown in purple, blue, and red, respectively. Cells in other regions are shown in grey. Red line indicates x=y.

**D.** Root mean squared error in scaling factors after each normalization method between the different region-skewed gene panels and the full gene panel.

**E.** Wilcoxon rank sum test -log_10_(p-values) adjusted for multiple hypothesis testing for one region-vs-all differential gene expression test with library size normalized gene expression from the ventricle (left), fiber tract (middle), and dentate gyrus (right) region-skewed gene panels versus the full gene panel. Purple, blue, and red indicates ventricle-, fiber tract- and dentate gyrus-vs-all tests, grey indicates other region-vs-all tests. Red line indicates x=y.

**F.** Differential gene expression false positive (left) and false negative (right) rates after each normalization method, taking differential gene expression results with the full gene panel to be ground truth, for each region-skewed gene panel.

**G.** One region-vs-all gene fold change with library size normalized gene expression from the ventricle (left), fiber tract (middle), and dentate gyrus (right) region-skewed gene panels versus the full gene panel. Purple, blue, and red indicates ventricle-, fiber tract- and dentate gyrus-vs-all comparisons, grey indicates other region-vs-all comparisons. Red line indicates x=y.

Black lines indicate 0 log_2_ fold change and grey lines indicate 0.5 log_2_ fold change.

**H.** Gene expression fold change switched positive (left) and switched negative (right) rates after each normalization method, taking differential gene expression results with the full gene panel to be ground truth, for each region-skewed gene panel.

**Figure S6. Impact of gene count normalization methods with region-skewed gene panels in seqFISH mouse kidney im-SRT data**

**A.** For a section of the mouse kidney assayed by seqFISH, heatmaps showing scaled mean gene expression of the 60 genes in 60-gene gene panel skewed towards cortex (left), medulla (middle), and pelvis (right) kidney regions for the three analyzed kidney regions.

**B.** Gene panel skew of all simulated and original full seqFISH gene panels.

**C.** Scatterplots of log_10_(scaling factors) with library size normalization for each cell for cortex (left), medulla (middle), and pelvis (right) region-skewed gene panels versus full gene panel. Cells in the cortex, medulla, and pelvis regions are shown in red, green, and blue, respectively. Cells in other regions are shown in grey. Red line indicates x=y.

**D.** Root mean squared error in scaling factors after each normalization method between the different region-skewed gene panels and the full gene panel.

**E.** Wilcoxon rank sum test -log_10_(p-values) adjusted for multiple hypothesis testing for one region-vs-all differential gene expression test with library size normalized gene expression from the cortex (left), medulla (middle), and pelvis (right) region-skewed gene panels versus the full gene panel. Red, green, and blue indicates cortex-, medulla- and pelvis-vs-all tests, grey indicates other region-vs-all tests. Red line indicates x=y.

**F.** Differential gene expression false positive (left) and false negative (right) rates after each normalization method, taking differential gene expression results with the full gene panel to be ground truth, for each region-skewed gene panel.

**G.** One region-vs-all gene fold change with library size normalized gene expression from the cortex (left), medulla (middle), and pelvis (right) region-skewed gene panels versus the full gene panel. Red, green, and blue indicates cortex-, medulla- and pelvis-vs-all comparisons, grey indicates other region-vs-all comparisons. Red line indicates x=y. Black lines indicate 0 log_2_ fold change and grey lines indicate 0.5 log_2_ fold change.

**H.** Gene expression fold change switched positive (left) and switched negative (right) rates after each normalization method, taking differential gene expression results with the full gene panel to be ground truth, for each region-skewed gene panel.

**Figure S7. Impact of gene count normalization methods with region-skewed gene panels in CosMx human liver im-SRT data**

**A.** For a section of human liver assayed by CosMx, heatmaps showing mean gene expression of the 200 genes in 200-gene gene panel skewed towards Zone 1 (left) and Zone 3 (right) regions for the two analyzed liver regions.

**B.** Gene panel skew of all simulated and original full CosMx gene panels.

**C.** Scatterplots of log_10_(scaling factors) with library size normalization for each cell for Zone 1 (left) and Zone 3 (right) region-skewed gene panels versus full gene panel. Cells in Zone 1 and Zone 3 are shown in red and cyan, respectively. Cells in other regions are shown in grey. Red line indicates x=y.

**D.** Root mean squared error in scaling factors after each normalization method between the different region-skewed gene panels and the full gene panel.

**E.** Wilcoxon rank sum test -log_10_(p-values) adjusted for multiple hypothesis testing for one region-vs-all differential gene expression test with library size normalized gene expression from the Zone 1 (left) and Zone 3 (right) region-skewed gene panels versus the full gene panel. Red and cyan indicates Zone 1 and Zone 3-vs-all tests, grey indicates other region-vs-all tests. Red line indicates x=y.

**F.** Differential gene expression false positive (left) and false negative (right) rates after each normalization method, taking differential gene expression results with the full gene panel to be ground truth, for each region-skewed gene panel.

**G.** One region-vs-all gene fold change with library size normalized gene expression from the Zone 1 (left) and Zone 3 (right) region-skewed gene panels versus the full gene panel. Red and cyan indicates Zone 1 and Zone 3-vs-all comparisons, grey indicates other region-vs-all comparisons. Red line indicates x=y. Black lines indicate 0 log_2_ fold change and grey lines indicate 0.5 log_2_ fold change.

**H.** Gene expression fold change switched positive (left) and switched negative (right) rates after each normalization method, taking differential gene expression results with the full gene panel to be ground truth, for each region-skewed gene panel.

**Figure S8. Impact of gene count normalization methods with region-skewed gene panels in 10X Xenium human breast cancer im-SRT data**

**A.** For a section of human breast cancer assayed by 10X Xenium, heatmaps showing mean gene expression of the 80 genes in 80-gene gene panel skewed towards invasive carcinoma (left) and ductal carcinoma *in situ* (DCIS, right) regions for the two analyzed regions.

**B.** Gene panel skew of all simulated and original full 10X Xenium gene panels.

**C.** Scatterplots of log_10_(scaling factors) with library size normalization for each cell for the invasive carcinoma (left) and DCIS (right) region-skewed gene panels versus full gene panel. Cells in the invasive carcinoma and DCIS regions are shown in cyan and red, respectively. Cells in other regions are shown in grey. Red line indicates x=y.

**D.** Root mean squared error in scaling factors after each normalization method between the different region-skewed gene panels and the full gene panel.

**E.** Wilcoxon rank sum test -log_10_(p-values) adjusted for multiple hypothesis testing for one region-vs-all differential gene expression test with library size normalized gene expression from the invasive carcinoma (left) and DCIS (right) region-skewed gene panels versus the full gene panel. Cyan and red indicates invasive carcinoma and DCIS-vs-all tests, grey indicates other region-vs-all tests. Red line indicates x=y.

**F.** Differential gene expression false positive (left) and false negative (right) rates after each normalization method, taking differential gene expression results with the full gene panel to be ground truth, for each region-skewed gene panel.

**G.** One region-vs-all gene fold change with library size normalized gene expression from the invasive carcinoma (left) and DCIS (right) region-skewed gene panels versus the full gene panel. Cyan and red indicates invasive carcinoma and DCIS-vs-all comparisons, grey indicates other region-vs-all comparisons. Red line indicates x=y. Black lines indicate 0 log_2_ fold change and grey lines indicate 0.5 log_2_ fold change.

**H.** Gene expression fold change switched positive (left) and switched negative (right) rates after each normalization method, taking differential gene expression results with the full gene panel to be ground truth, for each region-skewed gene panel.

**Figure S9. Spatially variable gene identification with full and region-skewed MERFISH gene panels**

**A.** nnSVG significant spatially variable gene expression test -log_10_(p-values) adjusted for multiple hypothesis testing with normalized gene expression from the full gene panel versus the habenula region-skewed gene panel. Red line indicates x=y.

**B.** nnSVG significant spatially variable gene expression test -log_10_(p-values) adjusted for multiple hypothesis testing with normalized gene expression from the full gene panel versus the fiber tract region-skewed gene panel. Red line indicates x=y.

**C.** nnSVG significant spatially variable gene expression test -log_10_(p-values) adjusted for multiple hypothesis testing with normalized gene expression from the full gene panel versus the dentate gyrus region-skewed gene panel. Red line indicates x=y.

**D.** Significant spatially variable gene expression false negative rates after each normalization method, taking significant spatially variable gene expression results with the full gene panel to be ground truth, for each region-skewed gene panel.

**Figure S10. Impact of gene count normalization methods with a non-skewed gene panels in MERFISH mouse brain im-SRT data**

**A.** For a coronal section of the mouse brain assayed by MERFISH, scatterplots of log_10_(scaling factors) for each cell based on different normalization methods (library size normalization, DESeq2 normalization, and TMM normalization) for non-skewed 100-gene gene panel versus the full gene panel. Cells within the ventricle, habenula, fiber tract, and dentate gyrus brain regions are shown in purple, green, blue, and red, respectively. Red line is x=y.

**B.** Root mean squared error in scaling factors after each normalization method between the different region-skewed and non-skewed gene panels and the full gene panel.

**C.** Pearson correlation coefficient (*r*) distribution across genes for normalized gene expression magnitudes from the full gene panel versus the non-skewed gene panel across different normalization methods evaluated.

**D.** Wilcoxon rank sum test -log_10_(p-values) adjusted for multiple hypothesis testing for one region-vs-all differential gene expression test with normalized gene expression from the full gene panel versus the non-skewed 100-gene gene panel. Purple, green, blue, and red indicate ventricle, habenula, fiber tract, and dentate gyrus region-vs-all tests, respectively. Red line indicates x=y.

**E.** Differential gene expression false positive (left) and false negative (right) rates after each normalization method, taking differential gene expression results with the full gene panel to be ground truth, for each region-skewed and non-skewed gene panel.

**F.** One region-vs-all gene fold change with normalized gene expression from the full gene panel versus the non-skewed 100-gene gene panel. Purple, green, blue, and red indicate ventricle, habenula, fiber tract, and dentate gyrus region-vs-all comparisons, respectively. Black lines indicate 0 log_2_ fold change, grey lines indicate 0.5 log_2_ fold change, red line indicates x=y.

**G.** Gene expression fold change switched positive (left) and switched negative (right) rates after each normalization method, taking differential gene expression results with the full gene panel to be ground truth, for each region-skewed gene panel.

**Figure S11. Quantification of gene panel skew for monocyte-skewed gene panels of increasing size simulated from single-cell RNA sequencing data from peripheral blood mononuclear cells**

**Figure S12. Simulation of impact of partial cell volume capture on differential expression testing after gene expression normalization**

**A.** Spatial position of two simulated cell subpopulations. Cell subpopulation A on average is positioned within the imaged Z planes (grey lines). Cell subpopulation B on average is positioned above the imaged Z planes.

**B.** Principal components of ground truth gene expression profiles of cell subpopulations A and B simulated using the splatter package with parameters estimated from the MERFISH mouse brain datasets. Gene expression profiles for cell subpopulations A and B are simulated with identical parameters.

**C.** P-values for differential gene expression testing between cell subpopulation A and cell subpopulation B. P-values are treated as ground truth for later evaluation.

**D.** Proportion of cell volume captured in imaged region based on simulated cell Z position.

**E.** Total cell gene counts captured based on proportion of cell volume captured.

**F.** Principal components of captured gene expression profiles of cell subpopulations A and B taking into account partial cell volume capture.

**G.** P-values for differential gene expression testing with simulated captured gene counts between cell subpopulation A and cell subpopulation B without normalization (left) and after library size, DESeq2, and cell volume normalization (right), compared to ground truth p-values.

**Figure S13. Impact of skewed gene panel and choice of normalization method.**

**A.** Skewed gene panels overrepresent the gene expression of specific tissue cell-types or regions. In a tissue with two cell-types, 1 and 2, group X genes are not differentially expressed between cell-types, and group Y and group Z genes are overexpressed in cell-types 1 and 2, respectively. Group Y includes more/more highly expressed genes than group Z making the gene panel skewed towards cell-type a.

**B.** Partial imaging of cells in cell-types results in undersampling cell gene expression. Cells A and B are of cell-type 1, where cell A is fully imaged and cell B is partially imaged. Cell C is of cell-type 2 and is fully imaged.

**C.** True differential expression relationships between cells A, B, C, given their cell-types. Group X genes are not differentially expressed between cells. Group Y genes are overexpressed in cells A and B compared to cell C. Group Z genes are overexpressed in cell C compared to cells A and B.

**D.** Cell library size (i.e. total detected counts) based on measured genes and accounting for sampling differences due to proportion of cell volume imaged.

**Bottom Left.** Detected gene counts and differential expression without normalization. Group X genes are undercounted in cell B due to partial cell volume imaging resulting in false positive DE when comparing to cells A and C. Similarly, Group Y genes are undercounted in cell B due to partial cell volume imaging resulting in false positive DE when comparing to cell A.

**Bottom Middle.** Volume normalized gene counts and differential expression with volume normalization. Volume normalization accounts for undersampling due to partial cell volume imaging. DE gene groups are correctly identified.

**Bottom Right.** Library size normalized gene counts and differential expression with library size normalization. Library size normalization incorrectly accounts for partial cell volume imaging. Normalized group X gene counts are inflated for cell C compared to cells A and B. This results in false positive DE for group X genes in cell C when comparing to cells A and B.

**Figure S14. Impact of non-skewed gene panel and choice of normalization method.**

**A.** In non-skewed gene panels, tissue cell-types or regions have similar total gene expression. In a tissue with two cell-types, 1 and 2, group X genes are not differentially expressed between cell-types, and group Y and group Z genes are overexpressed in cell-types 1 and 2, respectively. Group Y and group Z genes are expressed at similar magnitudes in their respective cell subpopulations making the gene panel non-skewed.

**B.** Partial imaging of cells in cell-types results in undersampling cell gene expression. Cells A and B are of cell-type 1, where cell A is fully imaged and cell B is partially imaged. Cell C is of cell-type 2 and is fully imaged.

**C.** True differential expression relationships between cells A, B, C, given their cell-types. Group X genes are not differentially expressed between cells. Group Y genes are overexpressed in cells A and B compared to cell C. Group Z genes are overexpressed in cell C compared to cells A and B.

**D.** Cell library size (i.e. total detected counts) based on measured genes and accounting for sampling differences due to proportion of cell volume imaged.

**Bottom Left.** Detected gene counts and differential expression without normalization. Group X genes are undercounted in cell B due to partial cell volume imaging resulting in false positive DE when comparing to cells A and C. Similarly, Group Y genes are undercounted in cell B due to partial cell volume imaging resulting in false positive DE when comparing to cell A.

**Bottom Middle.** Volume normalized gene counts and differential expression with volume normalization. Volume normalization accounts for undersampling due to partial cell volume imaging. DE gene groups are correctly identified.

**Bottom Right.** Library size normalized gene counts and differential expression with library size normalization. Library size normalization accounts for undersampling due to partial cell volume imaging. DE gene groups are correctly identified.
