# Supplementary material for: Gene count normalization in single-cell imaging-based spatially resolved transcriptomics
Source: Genome Biol. 2024 Jun 12;25:153. doi: 10.1186/s13059-024-03303-w (PMC11167774; doi:10.1186/s13059-024-03303-w)
Supplement: Supplementary file 2 — Additional file 2: Mathematical Note 1 [file 13059_2024_3303_MOESM2_ESM.docx]

**Mathematical Note 1:**

The impact of gene panel composition and count normalization method on downstream differential expression analysis can be demonstrated mathematically using the theoretical example below.

Suppose we are measuring gene expression in a tissue composed of two cell-types, A and B, with the following gene expression matrix:

$$G=\left( \begin{matrix} x_{A} & x_{B} \\ y_{A} & y_{B} \\ z_{A} & z_{B} \end{matrix} \right)$$

where the columns of $G$ denote the gene expression profiles of cell-types A and B, respectively, and where $x$, $y$, and $z$, denote the gene counts of groups of genes in cells of individual cell-types. Additionally, suppose that cells of cell-type A and cell-type B have similar cell volumes $v$.

If we consider gene expression data from 3 cells, $A$, $A'$, $B$, where cells $A$ and $A'$ are of cell-type A, and cell $B$ is of cell-type B, and cell $A'$ is partially captured with half of its cell volume imaged. The detected count matrix $G$ will be:

$$G=\left( \begin{matrix} x_{A} & \frac{1}{2}x_{A} & x_{B} \\ y_{A} & \frac{1}{2}y_{A} & y_{B} \\ z_{A} & \frac{1}{2}z_{A} & z_{B} \end{matrix} \right)$$

where the columns of $G$ are the detected counts for cells $A$, $A'$, and $B$, respectively, and the imaged cell volumes, $V$, will be:

$$V=\left( \begin{matrix} v & \frac{1}{2}v & v \end{matrix} \right)$$

**Defining gene panel skew:**

Gene panel skew, as defined in this study, can be determined by considering the relative number of counts detected for each cell-type. In a skewed gene panel, on average, total gene expression for cells of one cell-type is higher than that of other cell-types. In the above example, a gene panel is skewed if:

$$x_{A}+ y_{A}+ z_{A}\gg x_{B}+ y_{B}+ z_{B}$$

A skewed gene panel can include genes that are not differentially expressed, as well as differentially expressed genes that are markers for each cell-type (i.e. highly expressed in one cell-type and not others), provided that, on average, the total detected gene counts for cells of one cell-type is much greater than the total detected gene counts of cells of other cell-types.

Conversely, a gene panel is non-skewed if, on average, total detected gene counts are similar between cell-types. In the above example, a gene panel is non-skewed if:

$$x_{A}+ y_{A}+ z_{A}\approx x_{B}+ y_{B}+ z_{B}$$

**Gene count normalization in a skewed gene panel (Supplementary Figure 13):**

Consider a skewed gene panel where group $x$ genes are not differentially expressed between cell-types, and group $y$ and group $z$ genes are specific marker genes for cell-types A and B, respectively, i.e.:

$x_{A}\approx x_{B} \approx x$,

$y_{A}\gg y_{B}=0$,

$z_{B}\gg z_{A}=0$,

$$y_{A}\gg z_{B}$$

Detected gene counts $G_{o}$ for cells $A$, $A'$, and $B$ are:

$$G_{o}=\left( \begin{matrix} x & \frac{1}{2}x & x \\ y_{A} & \frac{1}{2}y_{A} & 0 \\ 0 & 0 & z_{B} \end{matrix} \right)$$

*No gene count normalization:*

Given these detected counts, if we were to compare gene expression for each pair of gene groups for each pair of cells (i.e. perform 1-vs-all differential gene expression) without any gene count normalization, we would erroneously conclude that:

- cell $A$ overexpressed group $x$ genes compared to cell $A^{'}$
- cell $B$ overexpressed group $x$ genes compared to cell $A^{'}$
- cell $A$ overexpresses group $y$ genes compared to cell $A'$

*Library size normalization:*

Given detected gene counts $G_{o}$, total detected counts (i.e. library size) for each cell is:

$$L =\left( \begin{matrix} x+y_{A} & \frac{1}{2}(x+y_{A}) & x+z_{B} \end{matrix} \right)$$

and library size normalized counts $G_{L}$ are:

$$G_{L}=\left( \begin{matrix} \frac{x}{x+ y_{A}} & \frac{x}{x+ y_{A}} & \frac{x}{x+ z_{B}} \\ \frac{y_{A}}{x+ y_{A}} & \frac{y_{A}}{x+ y_{A}} & 0 \\ 0 & 0 & \frac{z_{B}}{x+ z_{B}} \end{matrix} \right)$$

As specified above, this gene panel is skewed towards group $y$ genes, i.e. $y_{A}\gg z_{B}$ and therefore $\frac{x}{x+ y_{A}}\ll\frac{x}{x+ z_{B}}$. A 1-vs-all differential gene expression analysis of library size normalized counts between cells here would erroneously conclude that:

- cell $B$ overexpressed group $x$ genes compared to cell $A$
- cell $B$ overexpressed group $x$ genes compared to cell $A^{'}$

*Cell volume normalization:*

Given detected gene counts $G_{o}$ and cell volumes $V=\left( \begin{matrix} v & \frac{1}{2}v & v \end{matrix} \right)$, cell volume normalized counts $G_{V}$ are:

$$G_{V}=\left( \begin{matrix} \frac{x}{v} & \frac{x}{v} & \frac{x}{v} \\ \frac{y_{A}}{v} & \frac{y_{A}}{v} & 0 \\ 0 & 0 & \frac{z_{B}}{v} \end{matrix} \right)$$

Here, a 1-vs-all differential gene expression analysis of volume normalized gene counts would correctly conclude that:

- Group $x$ genes are not differentially expressed between cells $A$, $A^{'}$, and $B$
- Group $y$ genes are not differentially expressed between cells $A$ and $A^{'}$, but are overexpressed in cells $A$ and $A^{'}$ compared to cell $B$.
- Group $z$ genes are overexpressed in cell $B$ compared to cells $A$ and $A^{'}$.

**Gene count normalization in a non-skewed gene panel (Supplementary Figure 14):**

Consider a non-skewed gene panel where group $x$ genes are not differentially expressed between cell-types, and group $y$ and group $z$ genes are specific marker genes for cell-types A and B, respectively, i.e.:

$x_{A}\approx x_{B} \approx x$,

$y_{A}\gg y_{B}=0$,

$z_{B}\gg z_{A}=0$,

$$y_{A}\approx z_{B}$$

Detected gene counts $G_{o}$ for cells $A$, $A'$, and $B$ are:

$$G_{o}=\left( \begin{matrix} x & \frac{1}{2}x & x \\ y_{A} & \frac{1}{2}y_{A} & 0 \\ 0 & 0 & z_{B} \end{matrix} \right)$$

*No gene count normalization:*

As in the skewed gene panel case, not accounting for partial cell volume imaging and the resulting undersampling of detected gene counts results in false positive results on 1-vs-all differential gene expression where we would erroneously conclude that:

- cell $A$ overexpressed group $x$ genes compared to cell $A^{'}$
- cell $B$ overexpressed group $x$ genes compared to cell $A^{'}$
- cell $A$ overexpresses group $y$ genes compared to cell $A'$

*Library size normalization:*

As above, library size normalized counts $G_{L}$ are:

$$G_{L}=\left( \begin{matrix} \frac{x}{x+ y_{A}} & \frac{x}{x+ y_{A}} & \frac{x}{x+ z_{B}} \\ \frac{y_{A}}{x+ y_{A}} & \frac{y_{A}}{x+ y_{A}} & 0 \\ 0 & 0 & \frac{z_{B}}{x+ z_{B}} \end{matrix} \right)$$

Here, $y_{A}\approx z_{B}$ and therefore $\frac{x}{x+ y_{A}}\approx\frac{x}{x+ z_{B}}$. A 1-vs-all differential gene expression analysis of library size normalized counts between cells here would correctly identify that conclude that:

- Group $x$ genes are not differentially expressed between cells $A$, $A^{'}$, and $B$
- Group $y$ genes are not differentially expressed between cells $A$ and $A^{'}$, but are overexpressed in cells $A$ and $A^{'}$ compared to cell $B$.
- Group $z$ genes are overexpressed in cell $B$ compared to cells $A$ and $A^{'}$.

Notably, when $y_{A}\approx z_{B}$, cell library size is proportional to imaged cell volume such that normalizing by cell library size correctly accounts for differences in sampling due to incomplete cell capture.
